# Lyophilized and gamma-sterilized allogeneic bone implant used as a spacer for advancement of a modified tibial tuberosity in the treatment of cranial cruciate ligament disease in dogs

**DOI:** 10.1371/journal.pone.0220291

**Published:** 2019-08-05

**Authors:** Gláucia O. Morato, Artur G. Rocha, Denise G. Chung, Maria E. B. A. M. da Conceição, Bruno W. Minto, João G. Padilha Filho, Luis G. G. G. Dias

**Affiliations:** 1 Autonomus Veterinary Medical, Descalvado, São Paulo, Brazil; 2 Hospital Veterinário Universitário, Centro Universitário Central Paulista (Unicep), São Carlos, São Paulo, Brazil; 3 Division of Veterinary Surgery, Department of Clinic and Animal Surgery, College of Agricultural Sciences and Veterinary Medicine, São Paulo State University "Julio de Mesquita Filho", Jaboticabal Campus, São Paulo, Brazil; University of Bari, ITALY

## Abstract

The objective of this study was to evaluate the use of a lyophilized and gamma-sterilized allogeneic freeze-dried bone wedge as a spacer for advancement of a modified tibial tuberosity (mTTA) in 16 knees that were clinically diagnosed with cranial cruciate ligament disease. Patients underwent radiography before the surgical procedure as well as immediately after surgery and at 30, 60, 90 and 120 days post-surgery, and their locomotion was evaluated at the same time points except for the immediate postoperative period. The surgical wounds were evaluated for signs of infection and rejection of the bone implant. Locomotion was graded on a scale of 0–5, with 0 indicating no limping and 5 indicating limb functional impotence. The "tibial-tibial bone-tibial implant" interfaces were evaluated radiographically, and each interface was assigned scores of 0–3, with 0 indicating no contact between the implant and adjacent bone and 3 indicating a bone bridge throughout the interface. The patients showed good clinical and radiographic recovery. The lyophilized bone spacer allowed for easy storage and transport and rapid and satisfactory execution of mTTA while showing resistance to drilling and fixation with screws in 87.5% of cases and a mean surgical time of 45.9 minutes. No immunogenic reactions were observed in 93.7% of the cases. One patient presented infection of the surgical focus, which showed remission after antimicrobial therapy. All patients showed functional recovery of the operated limb, with the number of clinically healthy patients being higher than those with claudication at 120 days (p ≤ 0.05). In all patients, it was possible to verify the incorporation of the bone implant into the tibia. Bone union occurred progressively, and the degrees of bone union observed on radiographs at postoperative days 60, 90, and 120 were significantly greater (p < 0.05) than those observed in the immediate postoperative period and at 30 days.

## Introduction

Several techniques have been described for the treatment of cranial cruciate ligament disease in dogs [1,2]. Tibial osteotomies are the most recently recommended technique since they can facilitate changes in the knee geometry that can cancel out the force, that resulting in cranial translation of the tibia, thus ensuring dynamic stability of the joint [3–5].

Tibial tuberosity advancement (TTA) for treatment of knees with cranial cruciate ligament disease in dogs was introduced in the year 2002 and has shown excellent results, with limb function recovery achieved within a short postoperative period [5–7]. Subsequent studies have described successful modifications to the technique while ensuring results comparable to those obtained with the original technique [2,8].

The use of autogenous bone can reduce costs and has proven to be an excellent approach when employing a cage in TTA surgery, allowing bone repair and early recovery of limb function. However, a second surgical procedure is necessary for graft collection, which can increase the surgical time and postoperative morbidity [8].

Therefore, researchers are seeking alternatives that show the same excellent outcomes as those obtained with autogenous graft consolidation but do not result in additional morbidity. One such alternative is allogeneic grafting [9].

Therefore, this study proposed the use of a lyophilized and cortico-spongy allogeneic bone wedge as a spacer in modified TTA (mTTA) in dogs with cranial cruciate ligament disease. The study also aimed to evaluate the feasibility of using the allogeneic implant and assess local reactions to the implant for up to 120 days after surgery. In addition, the study attempted to radiographically demonstrate the possible benefits to bone consolidation resulting from the use of lyophilized bone as a spacer.

## Materials and methods

The protocols of this study were submitted for evaluation to the Ethics Committee on the Use of Animals (CEUA) of the Sao Paulo State Unversity—UNESP, Jaboticabal, and were approved under protocol number 07401/14.

### Bone implant preparation

For production of the bone bank used in this study, cortico-spongy bone samples were collected from the iliac crests of dogs of various breeds weighing over 15 kg that had been subjected to euthanasia after brain trauma. This procedure was performed after brain death, when there was no response to deep pain and spinal reflexes. The information about these dogs are in Table 1.

**Table 1:** Information about each giver dog that was submitted to euthanasia (age, breed, body weight, blood count).

Animal n°	Age (year)	Breed	Body weigh	White blood count
1	2	Mixed breed	31	12300
2	2	Labrador	39	15200
3	1	Boxer	36	9800
4	6	Great dane	62	10500

The euthanasia was performed with propofol (4mg/kg), followed by intravenous injection of potassium chloride 10% until there is no more heartbeat. After the euthanasia procedure, hair clipping and antisepsis were performed and surgical access to the dorsal pelvic region was obtained. Using an oscillatory saw, wedge-shaped, corno-spongy bone fragments with widths of 3, 6, 9, 12, and 15 mm were collected, as described by Rocha [8].

Periosteum and fibrous and muscular tissue adhering to the bones was removed. The samples were then washed with a high-pressure washer to remove any bone marrow contained in the spongy bone.

Next, the bone samples underwent a fat removal process using successive ethanol and chloroform baths (1:1 ratio) at 4°C.

The lyophilization process consisted of freezing in a freezer at -18°C, freezing in a freeze drier to a temperature of -60°C, and stabilization at a pressure of approximately 4 × 10^−1^ atm for three days.

The implants were packed in screw-top glass vials and sterilized by means of gamma irradiation at a dosage of 25 kGy. The material was kept at room temperature until the time of its use.

### Patients

This study was performed in 16 knees of 15 dogs with cranial cruciate ligament rupture who were attended to during the clinical-surgical routine at two University Veterinary Hospitals.

Patients with cranial cruciate ligament disease underwent physical examination, orthopedic examination, blood count (Table 1), and serum alanine aminotransferase (ALT) and creatinine measurements. Those who did not present any concomitant orthopedic disease or alterations in the physical and laboratory exams were included in the study. Animals were only included in this study if the owners agreed to the Terms of Free and Informed Consent.

### Surgical procedure

All the patients were underwent to arthrotomy to remove the rest of ligament and inspection of mensch, and meniscectomy was performed if meniscal lesion was confirmed, after that, the capsule was closed with poliglecaprone 25.

**Fig 1:**
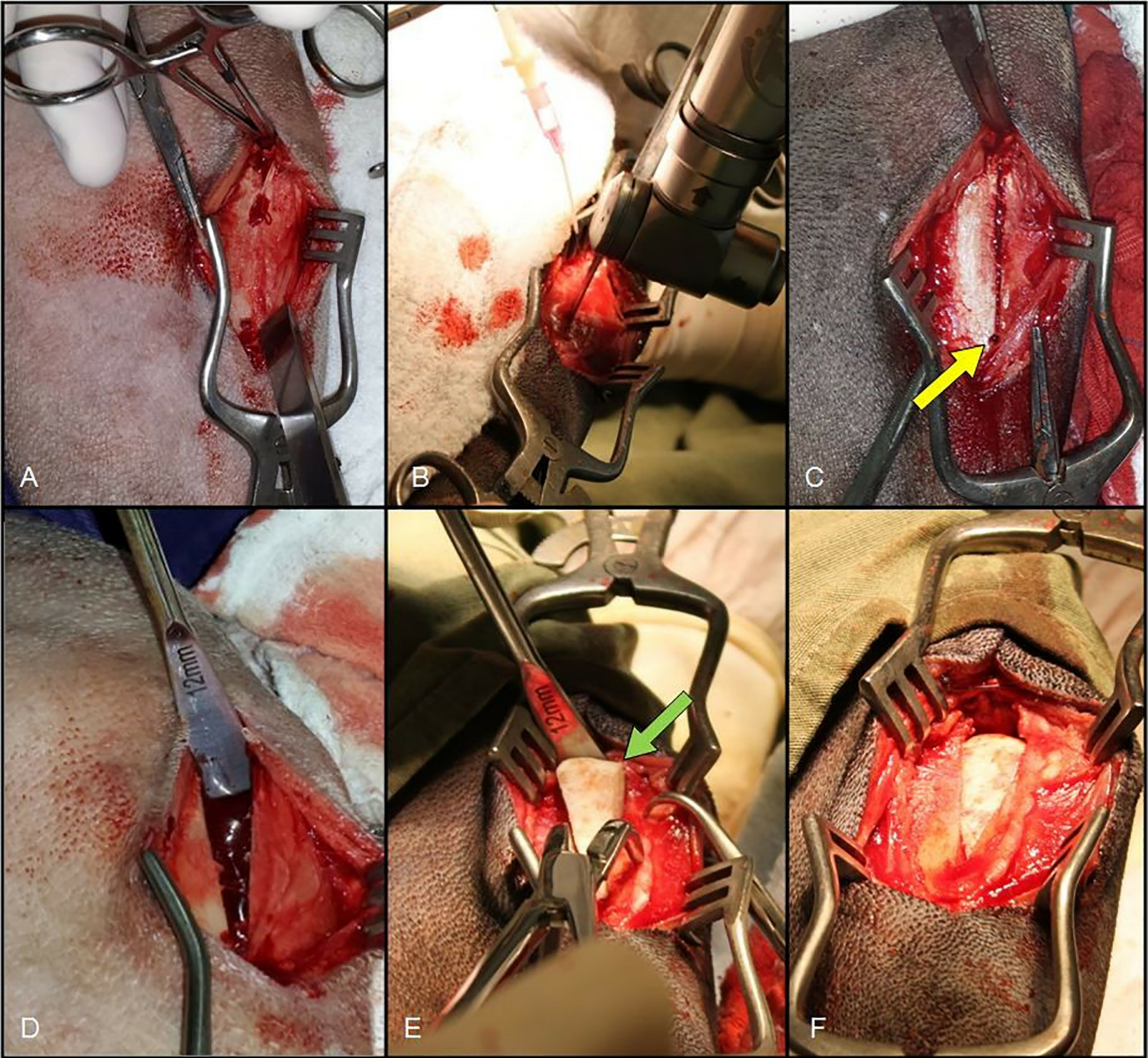
Image of the left pelvic member of a dog that underwent mTTA. Note the exposure of the medial face of the tibia after subcutaneous tissue dissection and the periosteum elevation, both with parsimony (A) to perform osteotomy immediately cranial to tibial plateau until to a distal hole in distal tibial tuberosity (B). In C, note the finished osteotomy line, which maintains the distal portion of the entire tibial tuberosity. The yellow arrow indicates the perforation made prior to osteotomy. In D, the tuberosity was removed and the gap was then filled with the lyophilized allogeneic cortical-spongy bone implant (green arrow, E and F).

Surgical access for mTTA was initiated with a cutaneous incision in the craniomedial aspect of the knee joint, extending from the tibial plateau to approximately 5 mm distal to the crest of the tibia. The subcutaneous tissue was revealed and the periosteum was elevated, both with parsimony, allowing exposure of the medial aspect of the proximal tibia (Fig 1A).

The patellar ligament was identified and isolated. The osteotomy line was determined immediately cranial to the tibial plateau, distally following a perforated hole in the final portion of the tibial tuberosity, which acted as both a distal reference point of the osteotomy and as a "hinge." The osteotomy was then performed with linear oscillatory sawing under irrigation with 0.9% NaCl solution (Fig 1B and 1C).

An allogeneic bone wedge was selected from the bone bank in accordance with the surgical plan. It was then immersed in 0.9% NaCl solution for at least three minutes. Using distractors, the tuberosity was removed from the body of the tibia (Fig 1D), and the resultant gap was filled with the bone implant (wedge) (Fig 1E and 1F).

**Fig 2:**
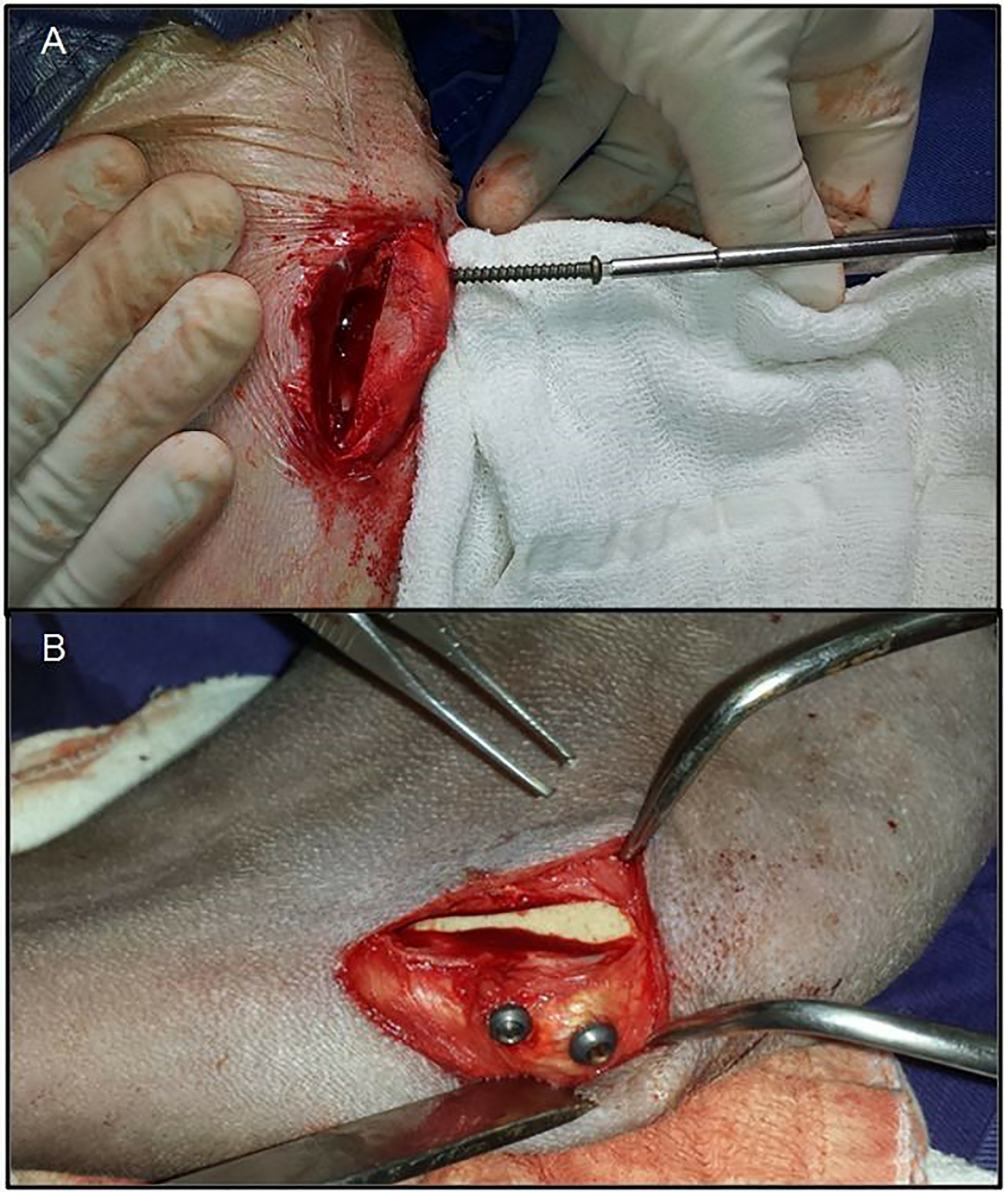
Fixation of the tibial crest and bone-cortico-spongy graft to the middle of the titanium screw in the craniocaudal direction.

Next, a hole (craniocaudal direction) was made immediately distal to the insertion of the patellar ligament and another hole was made about 5 mm distal to the first. After marking and measuring the depth of the holes with a cortical gauge, titanium screws of appropriate length and diameter ranging from 2.7 to 4 mm were inserted, thus fixing the tibial tuberosity, bone implant (wedge), and tibial body (Fig 2). Periosteum and subcutaneous tissue were sutured in a simple continuous pattern with poliglecaprone 25. The skin suture was performed with surgical nylon in Wolf stile.

The mTTA procedure was timed, starting from the skin incision to the last point of cutaneous synthesis.

Antimicrobial therapy with cephalexin (30 mg/kg) every 12 hours was prescribed for 10 to 15 days, analgesia with tramadol (4 mg/kg) and dipyrone (25 mg/kg) was administered every 8 hours for 10 days, and cleaning of the surgical wound was performed with NaCl 0,9%.

### Postoperative assessment

The surgical wound was evaluated to detect any tissue reaction at postoperative day 10 and at 30, 60, 90, and 120 days after the surgical intervention.

At the same periods, the presence or absence of secretions in the surgical wound and signs of exacerbated inflammatory reaction, such as edema, flushing, heat, and pain, were assessed visually and by palpation of the operative wound.

Functional recovery of the limb was evaluated by assessments of gait performed before surgery and at postoperative days 30, 60, 90, and 120. The assessment involved scoring the degree of lameness on the following numerical scale: 0 = absent, clinically healthy patient; 1 = absent to mild, detectable mild lameness; 2 = mild lameness; 3 = moderate claudication; 4 = moderate to severe, sparing the limb at trot; and 5 = severe, functional impotence [10]. Radiographs showing the mediolateral projections of the operated knees were obtained in the immediate postoperative period and at postoperative days 30, 60, 90, and 120. The union between the bone implant and the receptor site as well as the reabsorption of the implant were evaluated in these images.

Bone union was evaluated according to the method described by Minier et al. [11], in which scores of 0–3 were established for each of the interfaces (proximal and distal, or cranial and caudal) between the bone implant and receptor site, with the maximum and minimum total scores being six and zero, respectively.

In the present study, the bone/tibial crest interface was defined as the cranial interface and the bone/epiphysis implant interface of the tibia as the caudal interface. Resorption of the bone implant was evaluated qualitatively and described when present.

### Statistical analyses

All data were previously evaluated using Kolmogorov-Smirnov normality test. Gait and radiographic scores were not normally distributed. Thus, the different periods were compaired using Friedman’s test, followed by Dunn’s *post-hoc* test for multiple pairwise comparison. Confidence level adopted was 5%. Statistical analysis were performed using computer software GraphPad Prism 6 (Prism 4.00 –GraphPad Software).

For the other data obtained qualitatively, descriptive analyses were performed.

## Results

### Pre- and trans-surgical findings

A total of 16 knees of 15 dogs of different races, aged 1.2 to 12 years and with body weights ranging from 19 to 52 kg, were included in this study (Table 2).

**Table 2:** Information about each operated patient (age, breed, body weight, blood count).

Animal n°	Age (year)	Breed	Body weigh (Kg)	White blood cell count	Hematocrit (%)
1	2.5	English Bulldog	25.00	12300	43.8
2	9	American Bulldog	34.50	11900	41.2
3	5	Labrador Retriever	30.00	14300	47.5
4	7	Mixed breed	47.00	12900	47.3
5	6	Mixed breed	31.00	9800	40.3
6	9	Labrador	27.00	10200	39.1
7	1.2	Chow Chow	19.00	15100	50.4
8	3	American Pitbull	31.00	6700	52
9	6	American Pitbull	39.80	11900	49.4
10	8	American Pitbull	32.40	8500	40.5
11	4	Labrador Retriever	31.00	6500	45.2
12	4	Mixed breed	29.00	11600	46
13	2	Rottweiler	39.00	7000	47.6
14	3	American Pitbull	52.00	9100	42.3
15	12	American Pitbull	22.80	8800	36.8

During processing, the wedges (bone implants) showed greater palpable stiffness and more white coloration after lyophilization.

Sterilization by gamma irradiation did not macroscopically alter the bone implants; however, it caused changes in the color of the glass bottles, which were translucent before sterilization and turned amber after sterilization.

The use of bone bank implants provided great practicality and convenience in terms of storage and transportation. These allowed satisfactory execution of mTTA, presenting resistance to drilling and fixation by screws with diameters ranging from 2.7 to 4 mm in 87.5% of the cases (14/16 cases). In two procedures, the bone wedge fractured after the insertion of the screws and was then removed and replaced immediately with another wedge.

When positioned as a cage, the bone implant remained stable and under pressure between the tuberosity and the body of the tibia. When its placement in the formed gap was difficult, it could be quickly sculpted to ensure a better fit, which minimized the surgical time.

The mean surgical time was 45.9 minutes. The required implant widths were 12 mm in 75% of the cases and 9 mm in 25%; the lengths varied across cases.

In 5 (31.2%) of the 16 knees, arthrotomy and meniscectomy were performed, and in one of them, the tibial plateau was in an advanced stage of erosion, with a virtually degenerate meniscus./.

### Clinical assessment

Healing of the surgical wound occurred at 10 days, when the cutaneous suture was removed. No fistulas or intense inflammatory reactions were observed in 15 (93.75%) of the 16 operated knees. Only one patient presented with purulent secretion and infection of the surgical focus at 30 days, who presented with wet dermatitis and was treated with ciprofloxacin 10 mg/kg every 12 hours for 30 days. At postoperative day 60, it was possible to verify remission of the signs of infection (clinically and radiographically).

All the patients showed functional recovery of the limb and a return to satisfactory ambulation. The immediate postoperative period showed a worsening of the clinical picture, but it was followed by progressive recovery and a visually perceptible reduction in the degree of claudication over the follow-up period.

**Fig 3:**
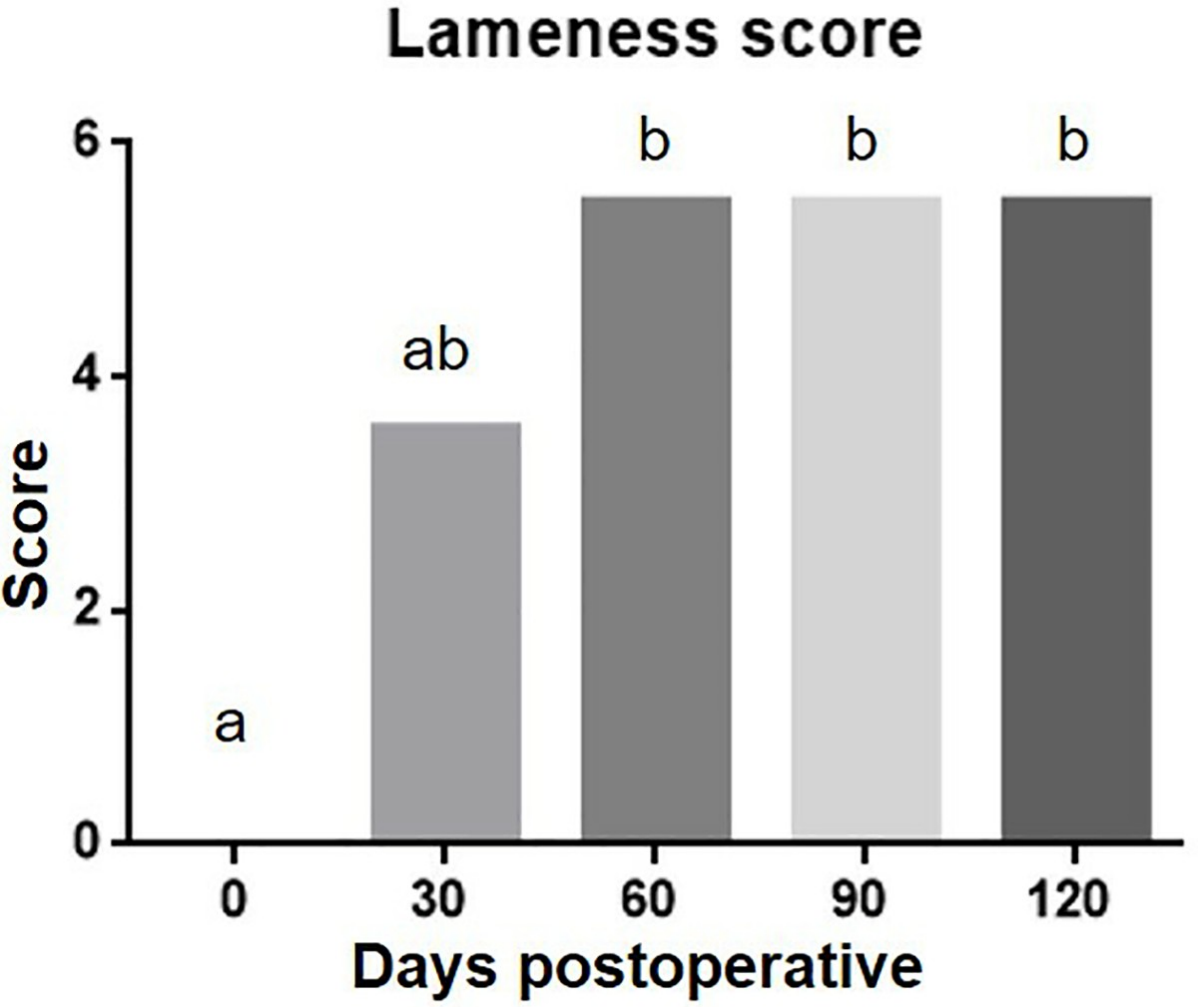
Column Graphic representing the lameness score median according to Quinn et al (10). The statistical analyzes was performed using non parametric friedman’s test and Dunn’s post test. It is observed that there was a progressive increase on the score, having a difference between preoperative period and postoperative (60, 90 and 120 days). Different letters represent significant difference (p>0.05).

In the preoperative period, the median score was significantly less than 60, 90 and 120 days postoperative (p< 0.0001). At 30 days postoperative, the lameness score was similar to the al periods evaluated, and at 60, 90 and 120 days were similar one each other (Fig 3).

**Fig 4:**
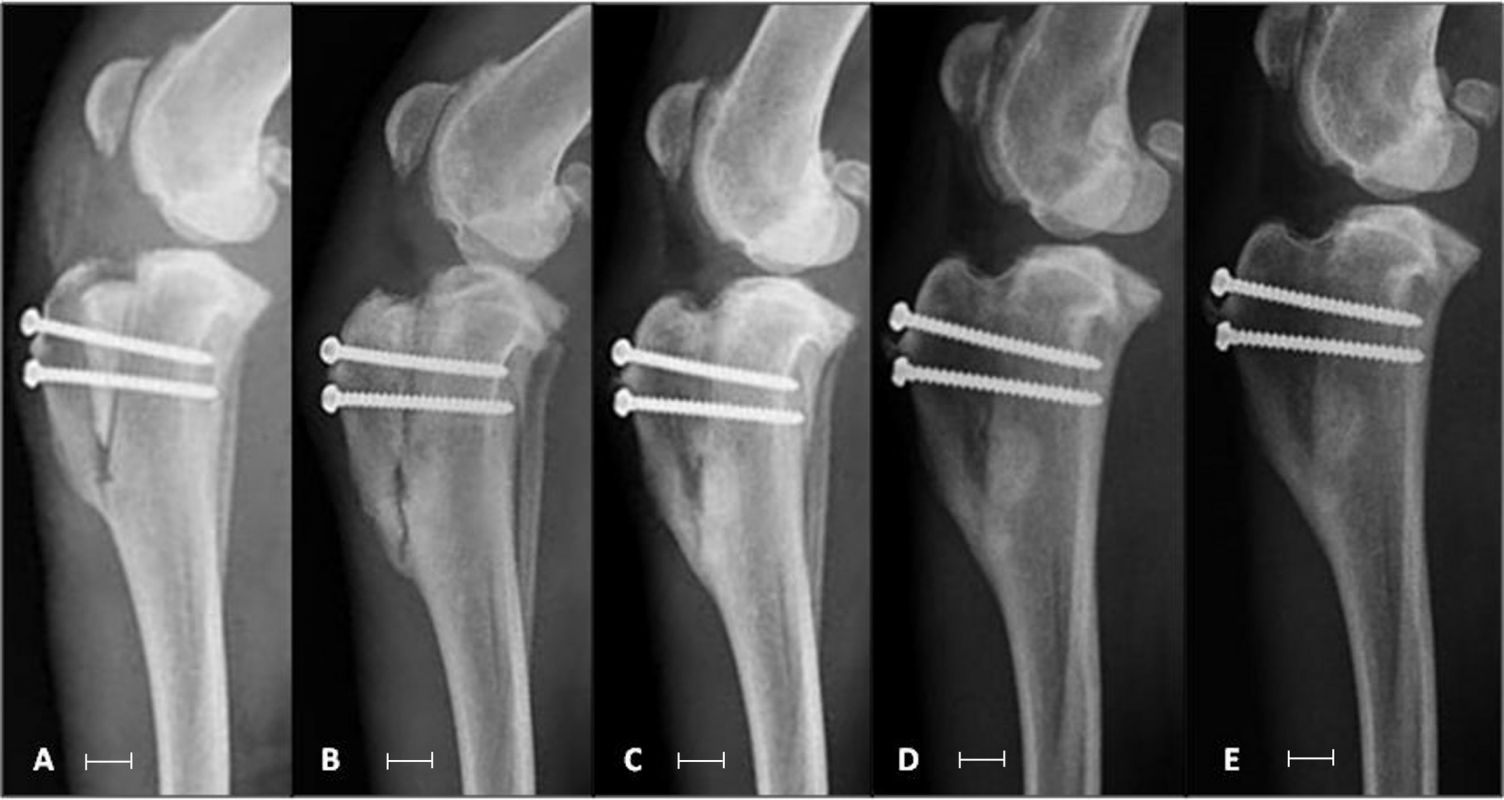
Radiographic images of the right knee of one patient (A-E) submitted to mTTA with the use of cortico-spongy bone as a spacer and fixation with titanium screws. Imidiatlly postoperative (A), at 30 (B), 60 (C), 90 (D) and 120 days (E). The white bars below the image has 1 cm length.

In all patients, incorporation of the bone implant into the tibia could be verified during the observation period (Fig 4) and maintenance of tibial crest advancement and positioning of the screws could be confirmed. None of the patients showed fracture or collapse of the implants, nor of the diaphysis of the tibia.

**Fig 5:**
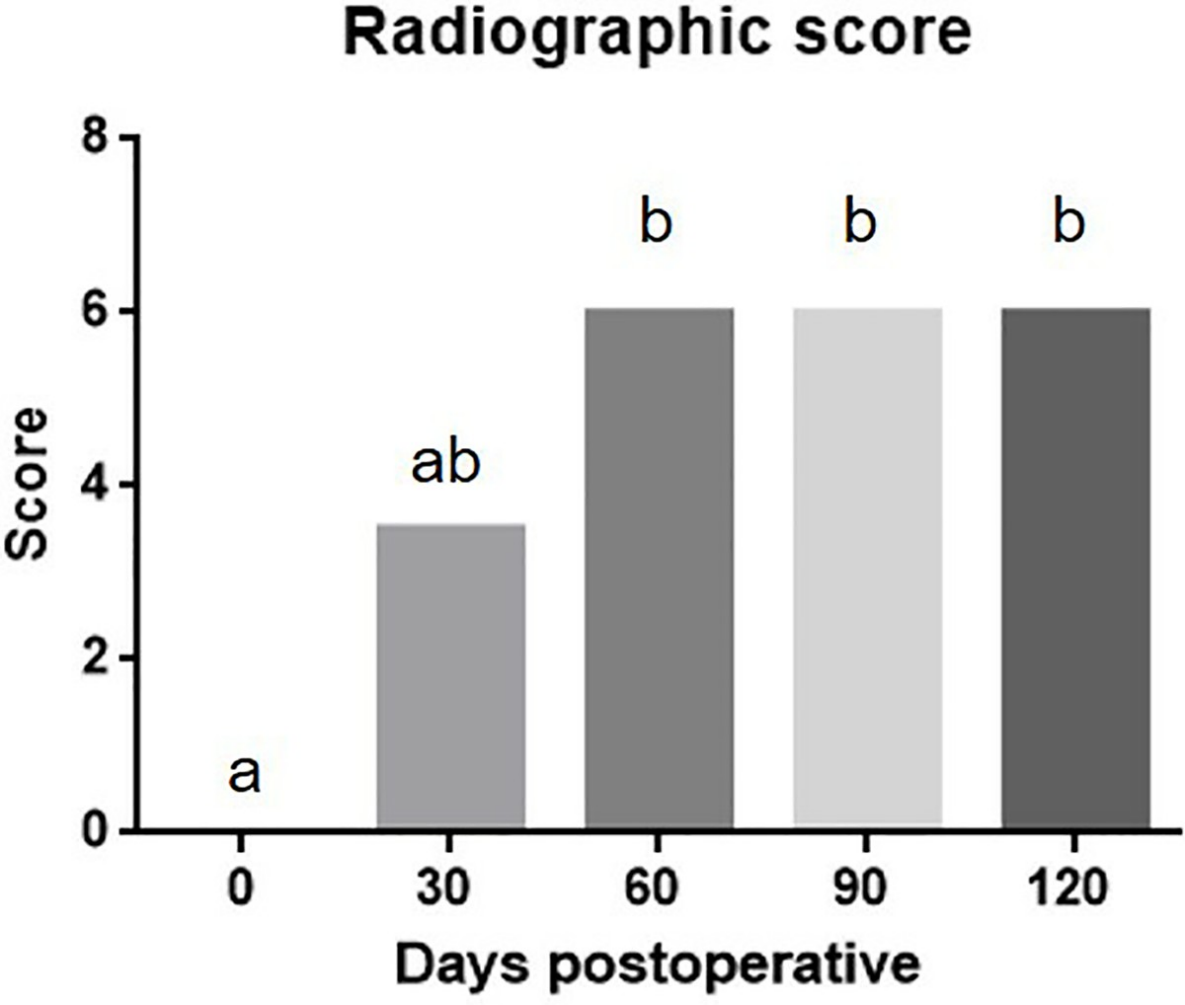
Column Graphic representing the incorporation of the bone implant into the tibia radiographic score median according to Minier et al (11). The statistical analyzes was performed using non parametric Friedman’s test and Dunn’s post test. It is observed that there was a progressive increase on the score, having a difference between preoperative period and postoperative (60, 90 and 120 days). Different letters represent significant difference (p>0.05).

Bone union occurred progressively, with the scores observed on radiographs at days 60, 90, and 120 being significantly higher (p< 0.0001) than those observed at immediate postoperative period (Fig 5).

Although the total maximum score remained similar (p ≥ 0.05) at 30, 60, 90, and 120 days, it was possible to visualize remodeling and bone organization during this follow-up period (Fig 5).

In 37.5% of the cases (6/16), fracture of the distal portion of the tibial crest was observed. In three cases, the fracture was observed in the transoperative period, when the tibial tuberosity was removed for positioning of the spacer, and in the remaining three cases, the fracture was observed in the radiographic examination performed at 30 days. However, the fracture was usually followed by evolution of bone consolidation without relevant clinical or radiographic consequences.

**Fig 6:**
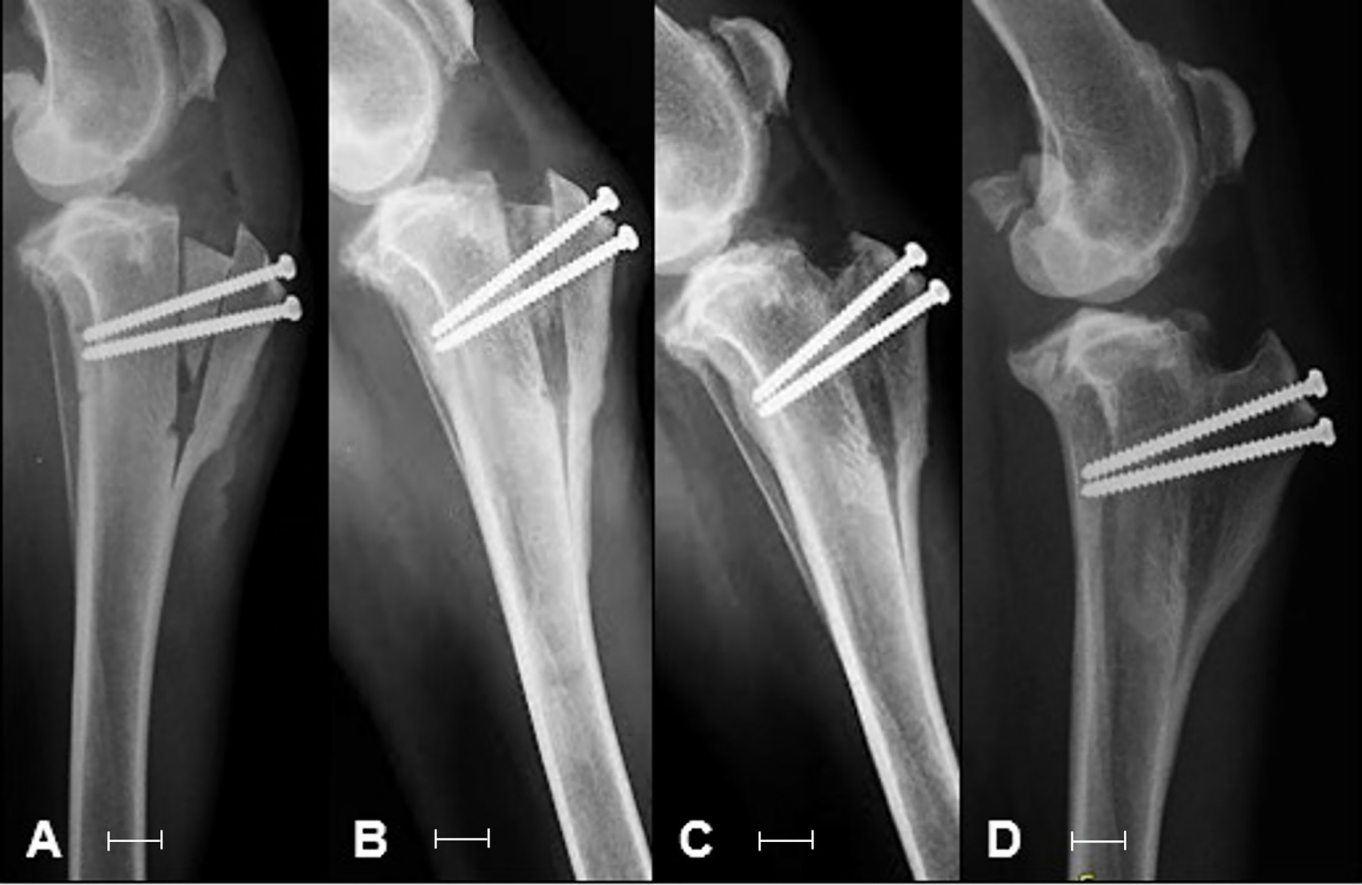
Radiographic images of the left knee of a dog submitted to mTTA using a cortico-spongy bone implant as a spacer and fixation with titanium screws. In B, 30 days after surgery, there was lysis and beginning of resorption of implanted bone due to infection of the surgical focus. After 60 days, after oral antimicrobial therapy, it is possible to verify bone union between implant and adjacent tissue, and in D (120 days), complete union without distinction between implant and adjacent bone. The white bars below the image has 1 cm length.

Implant resorption was observed in radiographic examinations of the patient who presented with purulent secretions at the surgical focus. In the image obtained at postoperative day 30, the distal portion of the bone wedge was reabsorbed. However, after initiation of antimicrobial therapy, evolution to bone consolidation was observed and there was no need to remove the implants to control infection (Fig 6).

## Discussion

While mTTA involves a 2–3-cm incision for bone implant positioning and fixation with screws [2,8], conventional TTA requires an incision of approximately 10 cm to allow placement and fixation of the plaque on the body of the tibia [6,7].

Since mTTA involves only two screws and uses the lyophilized bone implant as a cage, in comparison with the several specific implants required to perform conventional TTA, it is a simpler alternative technique. Additionally, it is important to note that in the case of mTTA, after complete bone healing, the screws can be easily removed from the tibia [12]. In addition, the titanium implant allows magnetic resonance examinations to be performed even while the equipment is present in the patient.

The patients in this study did not show foreign body-type reactions for up to 120 days after surgery. These observations allowed us to infer the low immunogenicity of the lyophilized bone, which corroborates the findings reported by Morato et al. [13], who noted no signs of rejection after xenogeneic implantation in rabbits of lyophilized canine bone sterilized using gamma irradiation. Additionally, Wang et al. [14], obtained similar results and, in histological assessments, observed that allogeneic lyophilized bone implanted in the reconstructed mandible of dogs over a 12-month period did not result in foreign body-type reactions in the patients, and was completely resorbed and replaced by neoformed bone.

The bone union observed radiographically between the cage and the tibia reinforces the claims in previous studies about the integration capacity of the lyophilized bone to the receptor site, which is directly related to the osteoconductive properties of this type of implant [15,16]. The radiographic union could be observed at 60 days postoperative, been similar to the exams at 90 and 120 days. However, when the use of lyophilized bone was compared in isolation with stem cells or autogenous bone marrow, it showed slower consolidation, probably due to the growth factors and osteogenic agents that added to the osteoconductive framework of lyophilized bone, accelerating the process of ossification [14].

The bone filling was slower in the distal portion of the gap. As the bone implant (cage) did not perfectly harbor this portion, this region may have shown impaired osteoconduction, since close contact and stability at the receptor site are essential for adequate cell migration between the bone implant structure and the receptor site [16,17].

The infection rate in this study was 6.25% (1/16), similar to that reported for TPLO by Gallagher and Mertens [18], but lower than the 13.46% value reported by Dal-Bó et al. [3] after performing three techniques (TTA, TPLO, and closing wedge osteotomy) for correction of CCL rupture. These authors attributed the higher rate of infection to the limited muscular coverage in the region of the implants, periosteal dissection, thermal necrosis during the use of the saw or perforator, and duration of anesthesia. This justifies the importance of irrigation during osteotomy as performed in this study, as well as the advantage of the shorter surgical time in comparison with conventional TTA.

Partial reabsorption of the graft in one patient was attributed to osteomyelitis, which manifested radiographically as points of osteopenia and partial resorption of the bone implant. According to Robinson [19], loss of organized bone structure frequently occurs in the inflammatory phase of osteomyelitis, leading to obliteration of the blood vessels of the bone tissue. The consequent ischemia after this process causes an increase in osteoclastic activity, resulting in necrosis and bone resorption.

The failure of antimicrobial therapy in cases of implant-related infections can be attributed to the decreased effect of the drug on the biofilm-protected microorganism [3,20]. In conventional TTA, removal of implants can be extremely complex due to the location of the cage within the osteotomy gap and the fork design that holds the plate in position [20,21]. In this study, it was possible to perceive that the use of the lyophilized bone implant instead of the cage favored control of the outbreak of infection with oral antimicrobial therapy, and no additional surgical interventions were necessary for the removal of implants, since the bacteria were incapable of remaining in a component that is reabsorbed or incorporated to the bone, which provided this cage an advantage over other types of cages [21].

The fracture of the distal portion of the tibial crest observed radiographically in three patients did not interfere with clinical and radiographic recovery. In a different context, Medeiros et al. [2] performed mTTA and found complications such as breakage and loosening of screws, in addition to infection of the surgical focus, which had to be managed by removing the cranial screws. Bander et al. [21] developed a cage of polyglycolic acid and metallic wings, and noted a very high complication rate of 38%, with the complications including cage wing break, plaque rupture, and surgical site infection, causing an increase in the time required for bone consolidation in the formed gap. These authors correlated the sagging of the cage wing and the consequent breakage of the plaque to the material used in the study.

All patients in this study showed functional recovery of the affected limb and gradual clinical improvement during the postoperative follow-up period, showing a normal gait at 60 days postoperative. Similar data have been reported in dogs with CCL rupture treated by conventional TTA [6,7] and mTTA [2,8,12], thus evidencing the positive results obtained using the biomechanical principle of TTA for functional limb recovery.

## Conclusion

We can conclude that canine cortico-spongiosum bone implants lyophilized and sterilized by gamma rays show low immunogenicity and can be used as spacers for mTTA in dogs, yielding osseointegration with the tibia and progressive clinical improvement in patients.

## Supporting information

S1 Table Lameness score assessment of each patient in each period of evaluation. (XLSX)

S2 Table Radiographic score assessment of each patient in each period of evaluation. (XLSX)
